# Depression, Anxiety and Their Correlates Among Patients With HIV in South Ethiopia: An Institution-Based Cross-Sectional Study

**DOI:** 10.3389/fpsyt.2019.00290

**Published:** 2019-05-07

**Authors:** Bereket Duko, Alemayehu Toma, Solomon Asnake, Yacob Abraham

**Affiliations:** ^1^Faculty of Health Sciences, College of Medicine and Health Sciences, Hawassa University, Hawassa, Ethiopia; ^2^Faculty of Medical Sciences, College of Medicine and Health Sciences, Hawassa University, Hawassa, Ethiopia

**Keywords:** depressive symptom, anxiety symptom, perceived stigma, social support, HIV, Ethiopia

## Abstract

**Background:** Depressive and anxious symptoms are more regularly seen in HIV-infected people than in the general population. This investigation planned to evaluate the magnitude and factors related to depressive and anxiety symptoms among HIV patients in South Ethiopia, 2018.

**Methods:** This was an institution-based cross-sectional study directed among 363 HIV-infected individuals who had a customary visit at Hawassa University Comprehensive Specialized Hospital and Yirgalem Hospital, Ethiopia, who were incorporated into the study through systematic sampling techniques. The hospital anxiety and depression scale (HADS) was utilized to take a look at anxious and depressive symptoms.

**Results:** The mean age of the respondents was 37.66 years (SD ±10.03). The prevalence of depression and anxiety were 32.0% and 34.4%, respectively. Patients who were living alone [AOR = 1.94, (95% CI: 1.06, 3.56)], had poor social support [AOR = 5.57, (95% CI: 1.20, 10.84)] or had HIV-related perceived stigma [AOR = 2.35, (95% CI: 1.44, 3.84)] were more likely to have depression as compared to their counterparts. Those with a previous history of mental illness [AOR = 3.36, (95% CI: 1.31, 8.61)] and poor social support [AOR = 6.67, (95% CI: 1.47, 10.33)] were more likely to have anxiety symptoms.

**Conclusion:** The prevalence of anxiety and depression in the current study was high. Concerned health departments of the country should create guidelines to screen and treat depression and anxiety among HIV patients. Further research on hazard factors of depression and anxiety ought to be examined to strengthen and expand these findings.

## Background

Human immunodeficiency virus (HIV) remains a noteworthy social issue worldwide in general and, in low- and middle-income nations specifically, where a considerable number of individuals living with HIV/AIDS (PLWHA) can be found. The WHO in 2017 reported that an estimated 36.7 million individuals were living with HIV infection and AIDS, with 2.1 million new cases and 1.1 million deaths as a result of HIV-associated causes (1).

Since 1990, HIV infection-associated death has reduced due to the introduction of active antiretroviral therapy (ART). Thus, people who are living with HIV/AIDS have begun to live longer. Nevertheless, people with HIV/AIDS are prone to mental illness, especially depression and anxiety, because of sexual-related problems, social and perceived stigma, the undesirable effects of antiretroviral treatment and neurophysiological changes (2, 3). Investigations indicated that as compared to HIV-negative individuals or the general population, depression occurs at rates two to four times higher in HIV-positive individuals (4–9). It has been observed to be related with higher HIV viral loads and lower CD4 number, even in the wake of controlling for the impacts of adherence, which predict illness advancement and mortality (6, 10–12).

Among psychiatric problems when compared with the general population, anxiety and depression are commonly found in HIV-affected people (6–10). Depression is a conceivably hazardous condition that can impact not just personal satisfaction, connections, work and adherence to therapeutic consideration, as well as possibly survival. The effect of mental health problems on HIV patients is frequently underestimated and is more critical in resource-constrained settings, which is due to an absence of training for health care providers, lack of awareness among HIV patients and lack of guidelines to manage psychiatric disorders in HIV clinics (13, 14).

People living with HIV/AIDS are increasingly inclined to display anxious and depressive symptoms which, thus, affects the stigma associated with the illness, decreases personal satisfaction, increases mortality, lessens medication adherence and impedes their capacity to resist disease (13–16). Having low income, being widowed, being female, having no job, substance abuse including alcohol, non-adherence to medication, low educational status and being in stage III and stage IV were factors that contribute to depression and anxiety among HIV patients (17–19). These show that anxiety and depression greatly affect these patient populations’ treatment outcomes.

Therefore, this study aimed to assess the magnitude and correlates of depressive and anxious symptom among HIV patients in South Ethiopia.

## Methods

**Study design and setting:** This research was undertaken as an institution-based cross-sectional study at Hawassa University Comprehensive Specialized Hospital (HUCSH) and Yirgalem General Hospital (YGH), South Ethiopia, from January 22, 2018 to March 22, 2018. HUCSH is the only comprehensive specialized university hospital in the region, and it is situated at Hawassa city, 273km from Addis Ababa, the capital of Ethiopia. This hospital started delivering service in 2004 and provides both outpatient and inpatient services for more than 18 million people in its catchment area. The hospital has over 400 beds for inpatient service. YGH is in the town of Yirgalem, which is 42km from Hawassa City and was established in 1966, delivers both inpatient and outpatient services to about 4.2 million people.

Sample size determination and sampling procedure**:** A single-population proportion formula was used to obtain the required sample size using a 95% confidence interval and a 5% margin of error using the prevalence of depression and anxiety: (larger proportion) proportion = 38.94% (17). Study participants were allocated to their respective study setting through a proportional allocation method. The study population was incorporated through a systematic sampling technique, K = 4. A total of 363 individuals with HIV who had follow-up for treatment were recruited for the study. The study participants who had hearing problems, patients who had known severe psychiatric illness or those who needed intensive care therapy were not interviewed.

**Data collection:** Experienced and trained psychiatry nurses gathered the data using interviewer-administered questioner. The data collection instrument incorporated socio-economic as well as demographic characteristics and clinically-related factors depicting questions. HIV-associated stigma was assessed through the 11-item HIV stigma scale. This scale comprised of four-point Likert questions concerning apparent isolation, shame, blame or guilt and disclosure of HIV status. The item scores of this scale questions summed to build a sole stigma variable. Study participants were classified as having or not having seen stigma utilizing the mean of the stigma scale (≥18.38 or ≥5.86) (20, 21). The Oslo 3-item social support scale was utilized to collect social support related issues. It has a total score scale running from 3 to 14 with three general classifications: “poor support” 3–8, “moderate support” 9–11 and “strong support” 12–14 (22). Anxious and depressive symptoms were assessed using the Hospital Anxiety and Depression Scale (HADS). This is a 14-item questionnaire used to screen for manifestations of depression and anxiety symptoms. It was approved for local use in Ethiopia, and its internal consistency was 0.78 for anxiety, 0.76 for depression subscales and 0.87 for both scales. The scales utilize a cut-off point for anxiety and depression >8 (23).

**Data Processing and Analyses:** The collected data was checked for comprehensiveness, consistency and, at that point data was coded, cleaned and entered into EPI info version 7. SPSS version 22 was utilized to examine the data. The association of every independent variable with the dependent variable was assessed by bivariate analysis. In order to distinguish potential confounders, a multi-variable logistic regression model was utilized. A p-value of under 0.05 was considered statistically significant, and adjusted odds ratio with 95% CI was determined to decide the association. Finally, the information was displayed by utilizing numbers, frequencies, tables, graphs and figures.

## Results

### Socio-Demographic Characteristics of the Study Participants

A total of 363 study participants were selected for the investigation with a participation rate of 98.1%. The mean (±SD) age of the respondents was 37.66 years (±10.03). Among the investigation participants, 239 (65.8%) were females, 165 (45.5%) had primary school as their maximum level of education, 188 (51.8%) were married, 128 (35.3%) were merchants and 128 (35.3%) received less than 2500 Ethiopian birr per month (Table 1).

**Table 1:** Socio-demographic characteristics of people living with HIV/AIDS at Hawassa University Comprehensive Specialized Hospital and Yirgalem Hospital, South Ethiopia, 2018.

Characteristics	Category	Frequency	Percent (%)
Sex	Male	124	34.2
	Female	239	65.8
Age	18–29	71	19.6
	30–39	147	40.5
	40–49	99	27.3
	≥50	46	12.7
Residence	Hawassa	213	58.7
	Yirgalem	150	41.3
Religion	Muslim	54	14.9
	Orthodox	155	42.7
	Protestant	122	33.6
	Catholic	32	8.8
Educational level	Unable to write and read	49	13.5
	Primary school	165	45.5
	High school	100	27.5
	Tertiary education	49	13.5
Marital status	Single	67	18.5
	Married	188	51.8
	Divorced	55	15.2
	Widowed/widower	53	14.6
Occupation status	Housewives	65	17.9
	Civil servants	81	22.3
	Privet employee/NGO	30	8.3
	Day laborer	27	7.4
	Merchants	128	35.3
	Unemployed	32	8.8
Monthly income	<2500 ETB per month	266	73.3
	2500–5000 ETB per month	78	21.5
	>5000 ETB per month	19	5.2

### Clinical and Psychosocial Characteristics of the Study Participants

A total of 309 (85.1%) of the study participants were on ART, 154 (42.4%) had poor social support, 308 (84.8%) had CD4 cell count ranges between 200 and 1000, 220 (60.6%) had a child or children and 45 (12.4%) were currently using substances (alcohol and tobacco products) (Table 2).

**Table 2:** Clinical and psychosocial characteristics of people living with HIV/AIDS at Hawassa University Comprehensive Specialized Hospital and Yirgalem Hospital, South Ethiopia, 2018.

Variables	Category	Frequency	Percent %
CD4 cell count	<200	29	8.0
	200–1000	308	84.8
	≥1000	26	7.2
On ART	Yes	309	85.1
	No	54	14.9
Perceived stigma	No	187	51.5
	Yes	176	48.5
Current substance	Yes	45	12.4
	No	318	87.6
Social support	Poor social support	154	42.4
	Moderate social support	179	49.3
	Strong social support	30	8.3
Family history of mental illness	Yes	43	11.8
	No	320	88.2
Previous history of mental illness	Yes	23	6.3
	No	340	93.7
Partner HIV status	Sero-positive	201	55.4
	Sero-negative	58	16.0
	Does not have partner	104	28.7
Have child/children	Yes	220	60.6
	No	143	39.4
Duration of illness	<5 years	11	30.6
	5–10 years	183	50.4
	≥10 years	69	19.0
Living status	With Family/relatives	284	78.2
	Alone	79	21.8
Co-morbid medical Illness	TB	38	10.5
	Diabetes	19	5.2
	Heart Diseases	16	4.4
	Renal diseases	14	3.9
	No co-morbid illness	276	76.0

### Prevalence of Depressive and Anxiety Symptoms and Their Correlates

The magnitude of co-occurring depression and anxiety in this study was 33.5%, while the prevalence of depression and anxiety was 32.0% and 34.4%, respectively. Multivariable binary logistic regression analysis revealed that HIV patients who had no children, were living alone, had perceived HIV related stigma and those who had poor social support were associated with depressive symptoms (Table 3). On the other hand, patients who had a previous history of psychiatric illness and poor social support were associated with anxiety symptoms (Table 4).

**Table 3:** Factors associated with depression among people living with HIV/AIDS at Hawassa University Comprehensive Specialized Hospital and Yirgalem Hospital, South Ethiopia, 2018.

Characteristics		Depression		COR (95% CI)	AOR (95% CI)
		Yes	No		
Sex	Male	90	34	1	1
	Female	157	82	1.38, (0.86, 2.23)	
Age	18–29	46	25	2.58, (1.04, 6.38)	
	30–39	91	56	2.92, (1.27, 6.72)	
	40–49	72	27	1.78, (0.74, 4.30)	
	≥50	38	8	1	1
Educational level	Unable to read & write	30	19	1.95, (0.82, 4.66)	
	Primary education	115	50	1.34, (0.64, 2.78)	
	Secondary education	65	35	1.66, (0.77, 3.59)	
	College and above	37	12	1	1
Marital status	Single	38	29	1	1
	Married	150	38	0.33, (0.18, 0.61)	
	Divorced	32	23	0.46, (0.46, 1.94)	
	Widowed/widower	27	26	1.26, (0.61, 2.60)	
Children	Have children	164	56	0.47, (0.30, 0.95)	0.53,(0.32, 1.16)
	Have no children	82	59	1	1
Living status	With family or relatives	210	74	1	1
	Alone	37	42	3.22, (1.92, 5.39)	1.94, (1.06, 3.56)**
Perceived stigma	Yes	144	43	2.37, (1.51, 3.74)	2.35, (1.44, 3.84)*
	No	103	73	1	1
Social support	Poor	89	65	10.23, (2.35, 14.46)	5.57, (1.20, 10.84)**
	Moderate	130	49	5.28, (1.21, 9.89)	3.75, (0.82,10.24)
	Strong	28	2	1	1

*Significant association (p-value < 0.05), **significant association (p-value < 0.01).

**Table 4:** Factors associated with anxiety among people living with HIV/AIDS at Hawassa University Comprehensive Specialized Hospital and Yirgalem Hospital, South Ethiopia, 2018.

Characteristics		Anxiety		COR (95% CI)	AOR (95% CI)
		Yes	No		
CD4 count	<200	20	9	0.64, (0.15, 2.84)	
	200–1000	198	110	0.79, (0.22, 2.88)	
	≥1000	20	6	1	1
Duration of illness	<5 years	76	35	1	1
	5–10 years	120	63	1.14, (0.68, 1.88)	
	≥10 years	42	27	1.39, (0.74, 2.62)	
Partner HIV status	Sero-positive	142	59	0.43, (0.26, 0.71)	
	Sero-negative	43	15	0.36, (0.18, 0.73)	
	Don’t have partner	53	51	1	1
Perceived stigma	No	124	63	1	1
	Yes	114	62	1.07, (0.69,1.65)	
Previous history of psychiatric illness	Yes	8	15	3.92,(1.61, 9.53)	3.36, (1.31,8.61)*
	No	230	110	1	1
Family history of mental illness	Yes	30	13	0.81, (0.40, 1.61)	0.65, (0.44, 1.24)
	No	208	112	1	1
Social support	Poor	83	71	11.98, (2.76, 22.04)	6.67, (1.47, 10.33)*
	Moderate	127	52	5.73, (1.32, 14.94)	3.83, (0.85, 8.23)
	Strong	28	2	1	1

*Significant association (p-value < 0.05).

## Discussion

The prevalence of depression in the current study was in line with finding from South Africa (24). However, the current study finding was higher than other studies in Ghana, Nigeria, South Africa and Brazil (18, 25–29). On the other hand, it is lower than other studies in Ethiopia (17, 26, 30), in Delhi (India) (19), North Central Nigeria (31), in Cameroon 63% (32), USA, Denmark (33, 34, China, India and Cameron (35–37). The prevalence of anxiety symptoms in the current study was 34.4%, which is similar with studies conducted in Ethiopia (25), USA, South Africa, Canada and Western Europe (17, 37–39). However, the finding was lower than studies conducted in Albania and China (19, 27, 32) but higher than studies conducted in Ethiopia, Ghana, Thailand, Brazil and Asia (26, 38, 40–42). This study used the hospital anxiety and depression scale (HADS) for assessing anxiety and depressive symptoms among HIV patients while others used the Hamilton depression scale (HDS), Beck’s depression scale (BDS), Beck’s anxiety scale (BAS), the State trait anxiety scale (STAS) or Patient health questionnaire item 9 (PHQ9). Socio-demographic and economic variation could play a vital role for the difference in the magnitude of depression and anxiety between studies from Ethiopia and other studies from other parts of the world.

Study participants who had HIV-related self-felt stigma had more depression when contrasted to their counterparts. This is in line with other findings in Botswana (43) and in Ethiopia (17, 26, 30, 34). HIV is associated with a large amount of stigma and, along these lines, HIV patients might think it is less demanding to be separated from everyone else in order to maintain a strategic distance from stigma or segregation, or they might not have the vitality to be socially connected (30–34). Stigma by itself might build dimensions of exhaustion and diminishing consideration or feelings of uselessness.

People living HIV/AIDS who were living alone were 1.94 times more prone to have depressive symptoms when contrasted to those HIV patients who were living with their family or relatives. Being forlorn is a solid hazard factor for depressive symptoms, well beyond proportions of target social interaction (44, 45).

Patients who had poor social support had a statically significant association with depressive and anxiety symptoms when contrasted to patients with good social support. This is comparable with other studies in India (19) and in Nigeria (18, 19, 22, 26, 31, 32). This may be because of the way that social separation diminishes social support, which can negatively affect mental and physical prosperity. Likewise, these patients preferred to abstain from looking for assistance from others and from opening up about their wellbeing because of social stigma towards themselves, which builds their seclusion and loneliness (19, 26).

Lastly, HIV patients with a past history of mental issues were 3.36 times more prone to experiencing anxiety symptoms. It is not clear whether the existence of HIV affects the seriousness of past psychiatric symptoms of patients or not. This may be because HIV by itself may cause progressively extreme symptoms. Moreover, HIV-affected patients with previous psychiatric problems presumably demonstrate a relapse of previous illness.

### Conclusion

The magnitudes of depression and anxiety among HIV patients were high (32% and 34.4%, respectively). Perceived HIV-related stigma, living alone and poor social support had a significant association with depressive symptoms. Having a previous history of psychiatric illness and poor social support had a significant association with anxiety symptoms. Concerned health departments of the country should create principles and standards to screen and treat these conditions in this patient population. Further research on the hazards of anxiety and depression ought to be directed to reinforce and widen these findings

### Study Limitations

We did not use standard tools or scales for substance abuse-related factors. Some important variables like medication adherence and opportunistic infections were not included.

### Ethical Approval and Consent to Participate:

Ethical clearance for this study was acquired from the Research and Ethics Review Committee of College of Medicine and Health Sciences, Hawassa University, Ethiopia. A letter of permission was acquired from the Research and Community Service Directorate of the College of Medicine and Health Sciences, Hawassa University and submitted to Hawassa University Comprehensive Specialized Hospital and Yirgalem General Hospital, Ethiopia. Study participants were informed about their rights to interrupt the interview at any time and written informed consent was obtained from each study participant. Confidentiality was maintained at all levels of the study. HIV-positive subjects who were found to have moderate to severe depressive and anxiety symptoms were referred to psychiatry clinics for further investigations.

## Data Availability Statement

All relevant data are within the paper.

## Ethics Statement

Ethical clearance for this study was obtained from the Research and Ethics Review Committee of the College of Medicine and Health Sciences, Hawassa University. Permission letter was obtained from Research and community service directorate of the College of Medicine and Health Sciences, Hawassa University and, submitted to Hawassa University Comprehensive Specialized Hospital and Yirgalem General Hospital. Study participants were informed about their rights to interrupt the interview at any time and written informed consent was obtained from each study participants. Confidentiality was maintained at all levels of the study. HIV positive subjects who were found to have moderate to severe depressive and anxiety symptoms were referred to psychiatry clinics for further investigations.

## Author Contributions

BD conceived the study and was involved in the study design, reviewed the article, analysis, report writing and drafted the manuscript. AT, YA and SA were involved in the study design and proposal development. All authors read and approved the final manuscript.

## Funding

This study was funded by Hawassa University, Ethiopia. The funding only covers the data collection and write up. No funding received for open access publications and other publication processing fee.

## Conflict of Interest Statement

The authors declare that the research was conducted in the absence of any commercial or financial relationships that could be construed as a potential conflict of interest.

The reviewer AT declared a shared affiliation, with no collaboration, with the authors to the handling editor.
